# Optimization of Power Utilization in Multimobile Robot Foraging Behavior Inspired by Honeybees System

**DOI:** 10.1155/2014/153162

**Published:** 2014-05-07

**Authors:** Faisul Arif Ahmad, Abd Rahman Ramli, Khairulmizam Samsudin, Shaiful Jahari Hashim

**Affiliations:** Department of Computer and Communication Systems Engineering, Faculty of Engineering, Universiti Putra Malaysia, 43400 UPM Serdang, Selangor, Malaysia

## Abstract

Deploying large numbers of mobile robots which can interact with each other produces swarm intelligent behavior. However, mobile robots are normally running with finite energy resource, supplied from finite battery. The limitation of energy resource required human intervention for recharging the batteries. The sharing information among the mobile robots would be one of the potentials to overcome the limitation on previously recharging system. A new approach is proposed based on integrated intelligent system inspired by foraging of honeybees applied to multimobile robot scenario. This integrated approach caters for both working and foraging stages for known/unknown power station locations. Swarm mobile robot inspired by honeybee is simulated to explore and identify the power station for battery recharging. The mobile robots will share the location information of the power station with each other. The result showed that mobile robots consume less energy and less time when they are cooperating with each other for foraging process. The optimizing of foraging behavior would result in the mobile robots spending more time to do real work.

## 1. Introduction

Today, research on multimobile robots using large numbers with biologically inspired system is increasing [1–3]. Multimobile robots consisting of more than one mobile robot are used in different environments and different types of mobile robot. The aim of multimobile robot application is applied to environment that is difficult or high risk work for human, especially in hazardous area and disaster area such as search and rescue in collapsed building. Currently, mobile robots are widely used in hazardous environment such as investigation in dangerous environment and exploration of nonhuman living environment such as space exploration and deep sea exploration. One of the examples was in Fukushima Daiichi nuclear power plant. The nuclear plant has been hit by disaster of earthquake and tsunami in east coast of Japan in year 2011 and caused the nuclear disaster to the nearest area. Mobile robots were used to explore the area of disaster in semiautonomous system [4].

In early day, several mobile robots with big size are used [5–8]. Nowadays, small size of body and simple mobile robots are very popular in research and education [1, 8–12]. Multimobile robot systems inspired by biological system such as ants, termites, honeybees, and fishes are defined as a swarm robot. Intelligence algorithm for control system which is inspired by biological system is defined as swarm intelligent system [13]. Swarm intelligence has been applied to homogeneous robotic system [11, 14] which is simple robots. But in [9, 15, 16], they used a group of various types of mobile robots which is defined as heterogeneous swarm robotic system. Most of the research on swarm mobile robotic system is

- focusing on communication systems among mobile robots [11, 15, 16] in order to share information,
- focusing on developed autonomous battery recharging [17–19] to have fully autonomous swarm robots without human intervention,
- focusing on developed navigation system [3] in order to run mobile robot without loss,
- focusing on developed management and optimization of energy system based on adaptation from biologically swarm behaviors [12, 19, 20],
- focusing on development of physical robot (small robot or macrorobot) [1, 11, 21] or simulated tools for swarm robotics [22–24].

Mobile robot is designed to be operated with finite energy resource, which is supplied by batteries. The energy of battery decreased with time due to robot processing, control system, and so forth. The battery power will run out if no action is taken to recharge it when it is running low. Researchers have developed an autonomous charging platform system for autonomous mobile robot systems. The first autonomous charging system on mobile robot is developed by Grey Walter in 1950, known as tortoise robot. Silverman et al. [18] had designed a docking platform for mobile robots recharging system. They defined threshold of battery voltage when robot needed to be recharged. A pan-tilt-zoom (PTZ) camera mounted on the mobile robot is used as a visual tool for searching orange colored piece of paper that is labeled above docking source. Cassinis et al. [25] developed a docking system for autonomous mobile robot charging system. Processing for docking operation system is implemented by creating a marker for reference by mobile robot during docking operation. The marker is identified by a vision system that is mounted on Pioneer 2DX robot. Ngo and Schiøler [26] developed a recharging system by exchanging the batteries. If a robot has low power battery during operation it will notify the coordinator robot (host) through the radio communication. The host will identify the nearest charger source or nearest robot that has high energy to the robot that requires energy. Then, the host will command the robot to go to nearest power station or command another robot that has high energy to go to the robot and exchange the battery. The message in communication is embedded with current position and status of the robot energy.

To operate mobile robot continuously, it needs to be recharged before its power resource is exhausted. The process of recharging battery needs to be fully autonomous; mobile robots need a capability of self-maintained or self-recharging battery system. In the previous paragraph, the researchers developed power station and function for autonomous recharging battery, but they did not identify how to handle the charging behavior with other behaviors such as robot task and robot interactions. Couture-Beil and Vaughan [27] applied the effect of charger location to evaluate system performance in limited environment. There were two environments that they examined which are location along robot's working path and nearby robot's working path. A minimum threshold of energy has been identified to drive the robot to the charging station. As the researcher limits the path environment of mobile robot with two ways of traveling, the time is increasing whenever numbers of mobile robots increased. Liu et al. [20] developed an autonomous ratio adjustment for several types of behaviors based on division labor from honeybees to maximize the net energy income to mobile robots. They used the assumption to identify the crowdedness of the foraging by assuming the frequent intersection of mobile robots. They did not study the relationship between number of power source and the number of intersection of mobile robot.

Several algorithms based on honeybee intelligent have been developed as optimization tools, such as artificial bee colony (ABC) [28] and honeybee mating optimization (HBMO) [29]. In the mobile robot application, both algorithms currently have been applied in the path-planning navigation [30, 31]. For example, in the ABC algorithm, four parameters have been divided, which are employed bee, onlooker bee, scout bee, and food source position. The division of bees in the environment will reduce the number of working robots in time. Based on that, the ABC algorithm is found not suitable to be applied to the mobile robots with working and foraging behavior that need to do task faster. Whereas in HBMO, the algorithm is based on finding the best result among the random execution of bee to find the optimal path. Another intelligent system from honeybee, which is based on foraging and working behavior, is more suitable to be applied to mobile robots having working and foraging power station as the main behavior.

In this paper, the work inspired by biological honeybees system is designed to optimize the working energy in the working behavior of mobile robots. In order to do that, the time utilization, power consumption, and traveling distance of the foraging behavior in the foraging area need to be decreased. Based on knowledge sharing and static threshold behavior, the system is applied to honeybees inspired environment and local communication.

A group of homogeneous mobile robots, known as AMiR [11], were used in simulation environment which is divided into two areas, which are home area and foraging area. This paper is divided to several sections.  Section 2 will discuss the background of autonomous recharging battery in mobile robots. In Section 3, the methodology of the development which is a swarm robotic system based on foraging behavior of honeybees is discussed and then continues with the experimental method. The result and discussion will be written in Section 4 and the conclusion in Section 5, respectively.

## 2. Background

In biological system, a swarm of honeybees shows that swarm intelligence and self-organisation task are done without centralized control decision [32], where each honeybee is capable of receiving inputs, makes its own decision, and then executes the decision by itself. In a beehive, honeybees worker is categorized based on different stages of age. The stage of honeybees has been identify as the honeybees worked from the first day of their life which is cleaning blood cells, tending larvae, hive construction, guarding the hive and foraging food [32, 33] (see Table 1).

**Figure 1:**
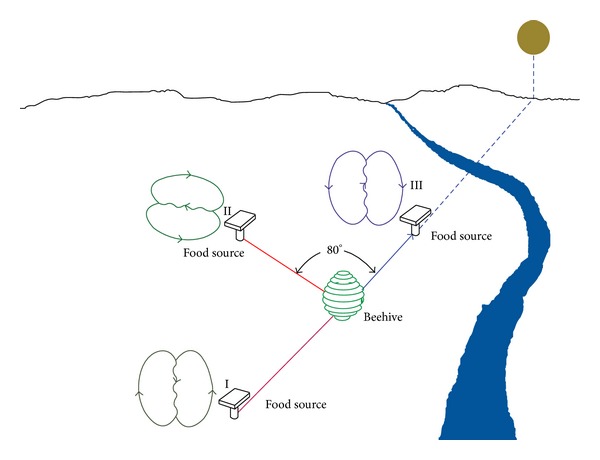
Honeybees communication through dances [32].

In biological honeybees, the information of the food source is shared with others through local communication. The information embedded with distance and direction of the food sources (I, II, III) is referring to beehive and the sun as shown in Figure 1. Two types of dances have been identified by von Frisch [34], which are waggle dance for longer distance and round dance for near distance.

Application of swarm intelligent system to mobile robots has been widely used with small or macromobile robots, such as swarm bot [14], autonomous miniature robot (AMiR) [11], EPUCK [21], JASMINE [35, 36], and ALICE [37]. Even though the size and the structure of mobile robot are small and simple, robotic system which is composed of large numbers can give better performance and robust compared to a complex and single mobile robot [38]. This type of mobile robot can be used in operation in small area such as a jet turbine and the complex engineering structure [10]. Another advantage is that the small size of robot can be easily and economically developed and replicated for being applied to bigger size of swarm robot. As the size is small, the processing power is also limited. But with large number of unit mobile robots that succeed by cooperation, it can overcome these disadvantages.

## 3. Methodology 

Mobile robots behavior was divided in two different behaviors which were working mobile robot for doing task in home area and foraging mobile robot for forage power station in foraging area. This behavior is inspired by the honeybees foraging and working system. For working behavior, mobile robots are walking around the home area in random way until their battery decreased to the threshold of remaining energy by percentage. For foraging mobile robot, it forages for power station randomly without remembering the path (Table 2). The following subsection describes the simulation platform and environment used in this work.

### 3.1. Simulation Environment

**Figure 2:**
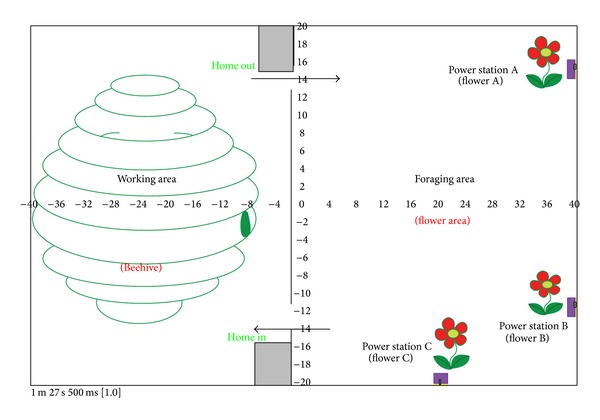
Environment for experiment of multimobile robots.

Inspired by behaviors of honeybees from their working stage, 10 mobile robots of AMiR are designed on the simulation platform which is known as Player/Stage [22].  Figure 2 shows the experimental environment with two separated areas, a working area (left side) and a foraging area (right side). The experimental environment imitated the environment of honeybees which work in a beehive and forage for food (honey) outside their home.

The foods (normally come from flower) of honeybees are allocated randomly outside area of their beehive. In the foraging area of mobile robot, three power stations which are power station A, power station B, and power station C are randomly placed in different locations (see Figure 2). All mobile robots have no information about the location of all power stations as also honeybees. Referring to Figure 2, working area (home area) and foraging area are separated by wall with doors as work area for biological honeybees is also separated from foraging area. The top door is provided for robot moving from working area to foraging area which is identified as home out (H(o)). The bottom door is used for mobile robot moving to working area from foraging area, identified as home in (H(i)). Mobile robots will be running randomly in home area and also searching for power station in foraging area when needed. Mobile robots started in the experiment with carrying energy in different capacities. Each of mobile robots has knowledge on exit and entry door for home area as honeybees have knowledge on their beehive's exit and entry. In the real environment, this could be implemented on office and house which normally have partition of rooms.

**Figure 3:**
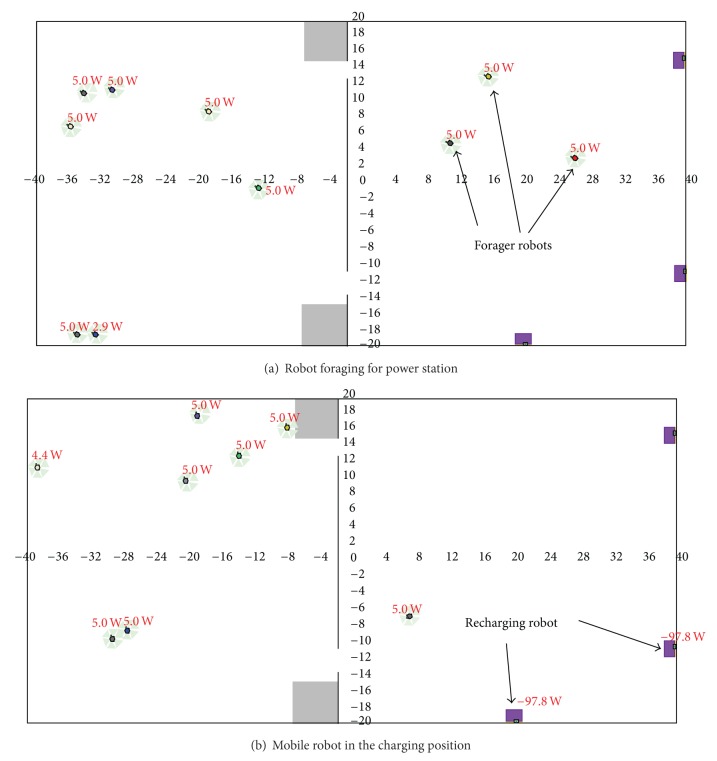
Some of mobile robots in stage of (a) foraging for power station (b) recharging at power station B and C.

**Figure 4:**
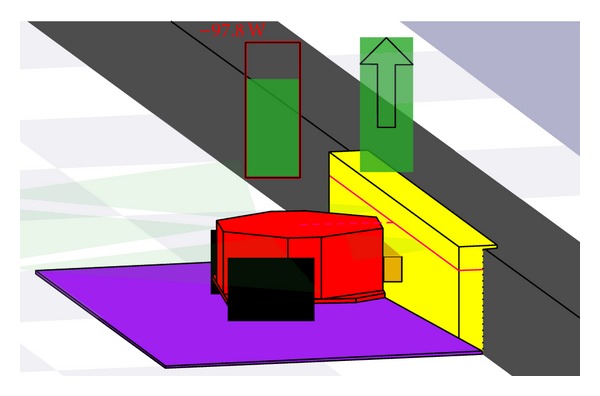
A closed view of mobile robots during charging at power station.

Figure 3 depicted foraging behaviors of multimobile robot in Player/Stage platform. Forager robot is moving randomly in foraging area (Figure 3(a)) looking for power station (A, B, or C). Whenever mobile robot finds a power station, it will identify the position of the location, remember it, and then charge until its battery is full (Figures 3(b) and 4).

**Figure 5:**
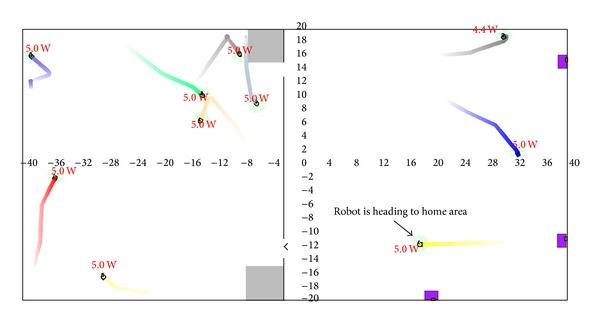
AMiR returning from charging station.

After the full charging, the mobile robot returns home through the entry door. The entry location is known by mobile robot as honeybees know the entry area of the beehive. The mobile robot used coordinate information as its guidance in the movement, as honeybees used their movement based on the reference of the beehive and the sun location (Figure 5).

**Figure 6:**
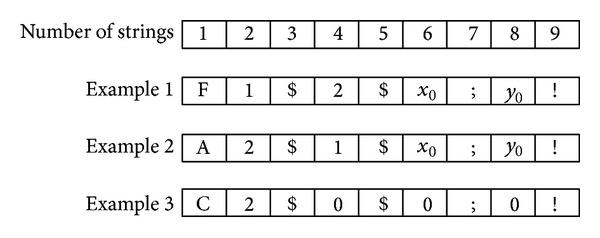
Message format in robots communication.

As in honeybees environment, the communication is done through the dance, which needs other nonknowledge honeybees face to face with the knowledge honeybees. Based on this, in mobile robots system, the movement of honeybees is converted to allocate the infrared (IR) surrounding the body of mobile robot. So the mobile robot does not need to move around during the communication, as location information is shared through infrared (IR) communication [39]. To adapt the IR communication in simulation platform, the UDP port is used as medium to transfer message between mobile robots as imitating an IR intercommunication in the real robot.  Figure 6 illustrates 9 character strings of message format. Each message is determined by availability of message, robots' identifications (robot ID), and location of power station.  Table 3 shows the detailed explanation of message format by each string. Example 1 is mobile Robot01 that detected power station by itself sending the message to mobile Robot02, while Example 2 is message that mobile Robot02 received information of location from Robot01 and lastly Example 3 shows initial message of Robot02 before it received the information from Robot01.

**Figure 7:**
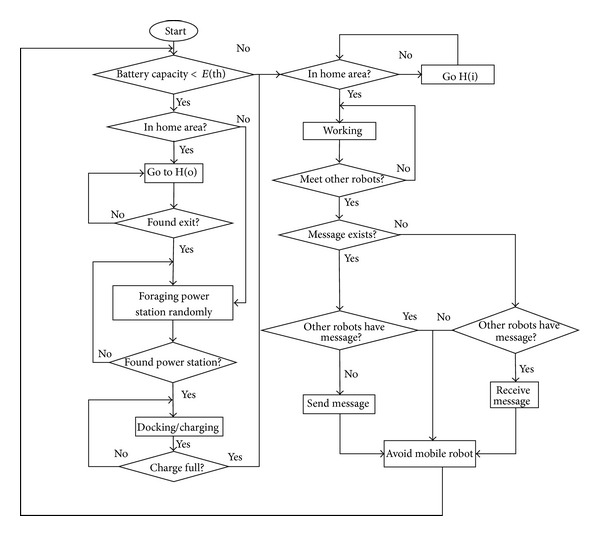
Algorithm of mobile robots behavior inspired by honeybees system.

Figure 7 shows the flowchart of algorithm in behaviors of multimobile robots during the foraging power station for charging their battery, sending and receiving message process, randomly moving in home area for working task, and also docking and undocking function to the power station.

In this work, the mobile robot that has work and change from working to foraging behavior is known as finishing one working iteration. After that, when it finished the foraging, recharged the battery, and moved back to home area, it is defined as finishing one foraging trip. Each of the working iterations or the foraging trips is measured by the performance metric.

### 3.2. Performance Metric

Three parameters are used to evaluate the foraging performance. The parameters are time consumption, power consumption, and traveling distance in foraging area. These three parameters are selected because

- time is determined by how long the mobile robot is foraging in the foraging area;
- power is determined by how much power is utilized in the foraging area. The assumption is that the mobile robot that utilizes little power in the foraging area will utilize more power in the working area. This means that mobile robot can do more work based on the more power that has been decreased;
- distance is determined by how long the distance can mobile robots take for their foraging. The short distance in the foraging behavior will result in the mobile robots consuming less time, less power consumption in the foraging area.

Foraging time, *t*, is measured as shown in (1) where *t*_exit_ is the time that mobile robot reaches H(o) and *t*_charge_ is time that mobile robots start to charge at the power station. The time unit is second (s). Consider

(1) $$\begin{array}{c} t_{\text{forage}}=t_{\text{charge}}-t_{\text{exit}}\left(0\right). \end{array}$$

Power consumption for foraging (*P*_forage_) mobile robot is measured by (2), where *P*_charge_ is the remaining of power battery at the time that mobile robot reached the power station and *P*_exit_ is the remaining of power battery at H(o) position. Power is measured with unit Joule (J). Consider

(2) $$\begin{array}{c} P_{\text{forage}}=P_{\text{charge}}-P_{\text{exit}}. \end{array}$$

**Figure 8:**
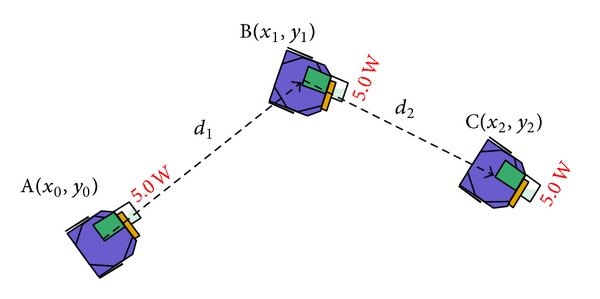
Traveling distance of mobile robot.

Finally, the traveling distance by foraging robot *n*, *D*_*n*_, is calculated using (3). Each of the iterations *i* of distance, *d*_*i*_, is defined based on two points before the rotation angle is changed as equation (see Figure 8 for the illustration). Consider

(3) $$\begin{array}{c} D_{n}=\overset{}{\underset{}{\sum }}d_{i},\\ d_{i}=\sqrt{{\left(y_{1}-y_{0}\right)}^{2}+{\left(x_{0}-x_{1}\right)}^{2}}. \end{array}$$

## 4. Result 

**Figure 9:**
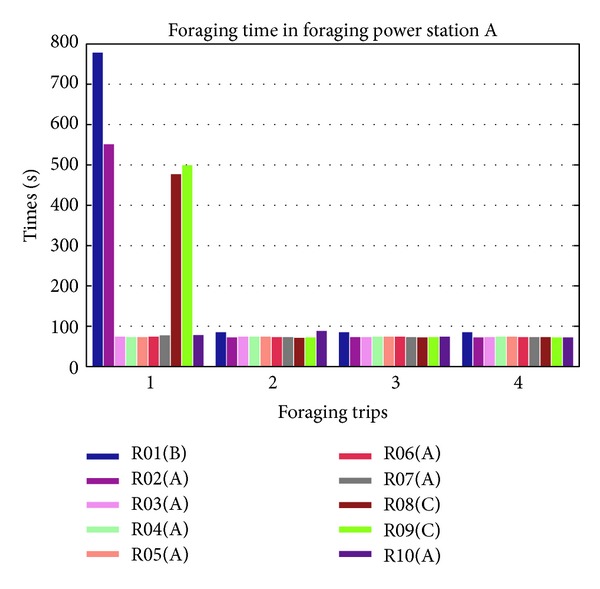
Time consumption in the foraging behavior of 10 mobile robots to power stations A, B, and C.

**Figure 10:**
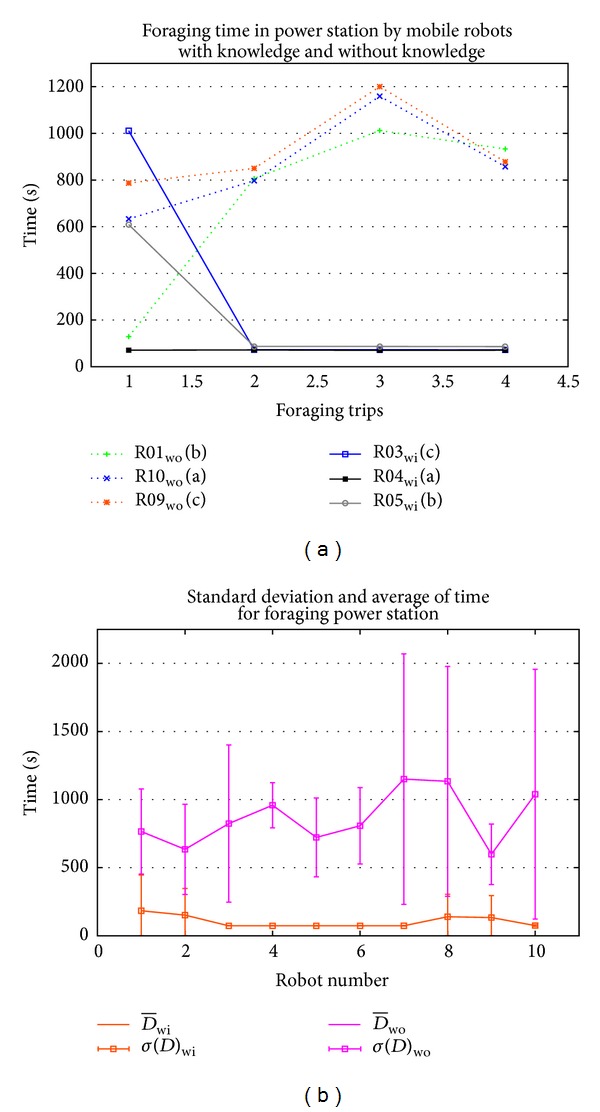
Analysis of foraging time consumption in environment with knowledge sharing and without knowledge sharing based on (a) 3 mobile robots in each environment and (b) average and standard deviation of times consumption for 10 mobile robots.

The results of time consumption in the foraging behavior for power station are shown in Figure 9. Mobile robots with ID R01, R02, R08, and R09 consumed a lot of time to forage power station during the first foraging trip. After the first foraging trip, the mobile robots shared the locations information with other mobile robots such as R03 and R04. With this information, mobile robots will consumed less time to reach power station. The difference of the time consumption for mobile robot without information compared to mobile robot with information is quite large which is almost 6 times larger as shown in Figure 10(a).

During the first foraging trip, R01, R02, R08, and R09 consumed a lot of time because the mobile robots need to forage power station randomly without information. They needed to forage until they found the station. The other robots with information did not consume a lot of time because they were heading directly to the power station without random forage. This difference is dynamic, because mobile robots forage in random way and without memorizing their path. This is proved by the result in the first trip of foraging mobile robots R01, R02, R08, and Robot09 which foraged to power stations A, B, and C (see Figure 9).

Figure 10(a) shows the comparison of the foraging time mobile robots with and without knowledge. R01, R09, and R10 are mobile robots that forage without information to the power stations A, C, and B, respectively. It is shown that the time consumption of mobile robot without knowledge is much longer than for the mobile robots that forage to power station with knowledge sharing. All the foraging was done in random way; R10 suddenly got high value in the third foraging trip compared to others. This is different with mobile robots that forage with the knowledge sharing environment, such as R03, R04, and R05, which forage with low value of time consumption beginning from the second foraging trip. When the time consumption is low in the foraging behavior, the time for mobile robots to do their task is increased. In that case, mobile robot should have much time to do task and work. These matters will make the task complete faster or earlier.

Figure 10(b) shows the average and standard deviation of foraging time by 10 mobile robots with sharing and without sharing knowledge. In environment of sharing knowledge, R01, R02, R08, and R09 show the high variance compared to other mobile robots. The mobile robots forage without knowledge in the first foraging trip and then memorized the information of the location power station. The average of time taken by mobile robots that forage for power station with information is less than 100 s, and their variance is very small compared to the mobile robots that forage without information at the first foraging trip. Meanwhile the mobile robots in environment without sharing knowledge show that the average of foraging is more than 600 s. The variance is also high compared to the mobile robots with sharing knowledge environment.

**Figure 11:**
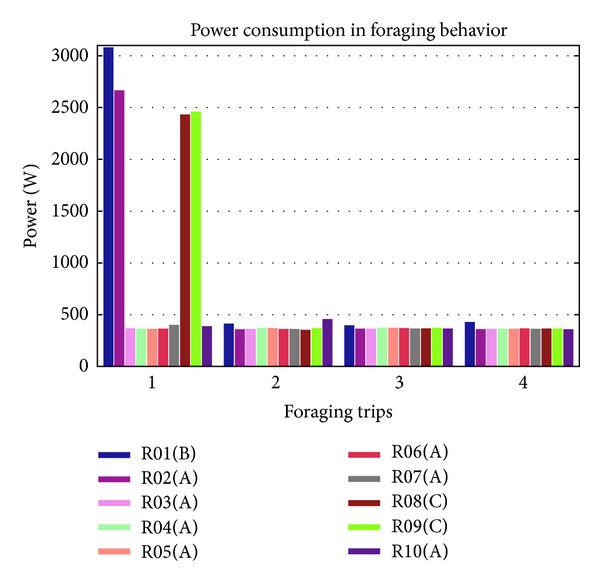
Foraging power consumption.

Figure 11 shows the power consumption of foraging power station by four foraging trips. The graph shows a lot of differences in foraging with knowledge and foraging without knowledge during the first foraging trip. As shown in the figure, R02 consumed high power to forage power station A during the first foraging trip because it does not have knowledge of the location. The mobile robot had foraged randomly until it found the power station and then memorized its location and in the next foraging trips the robot consumed less power. R02 not only memorized the location for itself but also shared the knowledge among other mobile robots such as R03, R04, and R05, so that other mobile robots do not need to forage and consumed a lot of power to do recharging.

**Figure 12:**
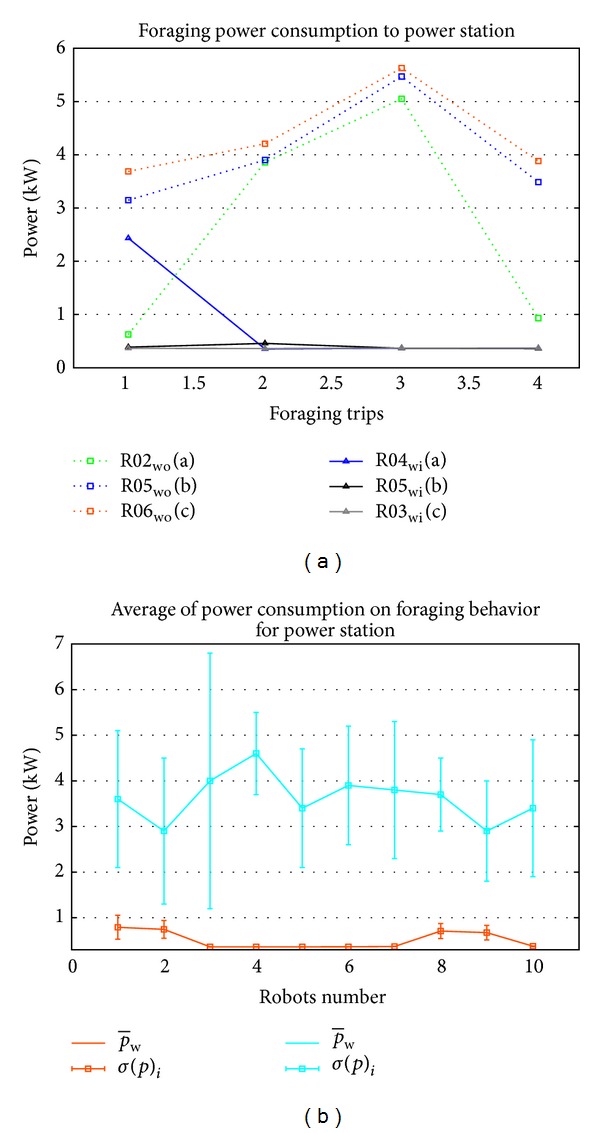
Analysis of foraging power consumption in environment with knowledge sharing and without knowledge sharing by (a) 3 selected mobile robots in each environment and (b) average and standard deviation of power consumption in foraging behavior.

Mobile robot with ID R01, R08 and R09 are consumed high power only at the first foraging trip, and then consumed low power for following trips with the knowledge. Among 10 mobile robots, in average R01, R02, R08, and R09 consumed high power in the foraging behavior. From the results of power consumption in foraging with knowledge sharing, three samples have been taken to compare with other three mobile robots that forage without knowledge sharing as shown in Figure 12(a). Mobile robots with ID R04 in environment with knowledge sharing show that they only consumed high power in the first trip, and other R03 and R05 consumed less power starting at the first foraging trip which is less than 1 kW, while for mobile robots that forage in environment without knowledge sharing, the power consumption is high which is more than 3 kW.

The average of power consumption in the foraging mobile robots with knowledge sharing and without knowledge sharing is shown in Figure 12(b). In without knowledge sharing case, the lowest value of power consumption is R03 and R09 with 2.9 kW. The maximum value is R04 with 4.6 kW. Robots with ID R01, R02, R08, and R09 consumed high power during foraging which is more than 650 W, while other mobile robots such as R03, R04, and R06 consumed less power which is less than 400 W. This happened because, during the first foraging trip, R01, R02, R08, and R09 need to forage in random way without the knowledge. But other mobile robots received the knowledge of the location power station before they changed to foraging behavior. In standard deviation the high variance of power consumption occurred on mobile robots that forage without knowledge in first foraging trip.

**Figure 13:**
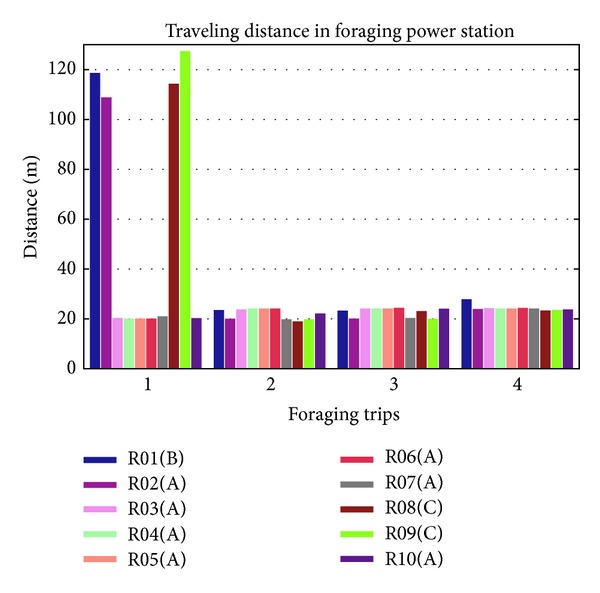
Traveling distance of 10 mobile robots on foraging behavior to power stations A, B, and C.

The result of traveling distance in foraging behavior for power station is depicted in Figure 13. Mobile robots with ID R01, R02, R08, and R09 traveled much longer than other mobile robots in the first trip as they are foraging without knowledge. But the other mobile robots such as R03 and R04 got the knowledge of the location on power station A from R02 and consumed shorter distance.

**Figure 14:**
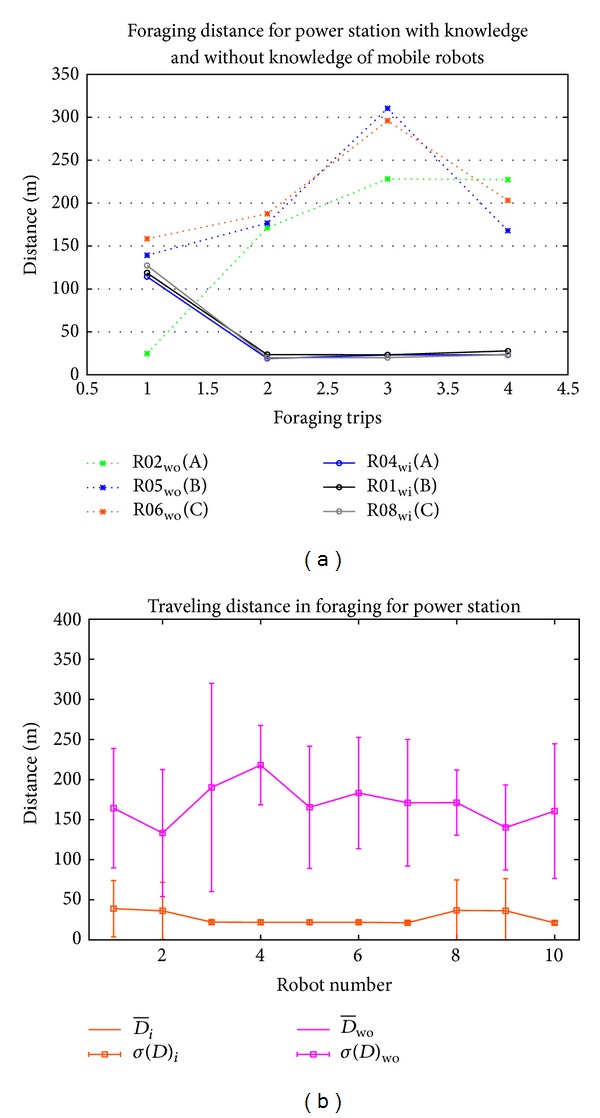
Traveling distance in foraging behavior with knowledge sharing and without knowledge sharing by (a) selected 3 mobile robots of each environment and (b) average and standard deviation of traveling distance in foraging for power station.

Figure 14(a) shows the analysis of traveling distance by choosing three mobile robots that forage with sharing knowledge and another three mobile robots without sharing knowledge. Mobile robots without knowledge sharing show long distance in traveling. Only R02 foraged in the first foraging trip with short distance but then foraged with long distance in the following foraging. Meanwhile, in environment with knowledge sharing, the short distance traveling is taken by mobile robots starting from second foraging trip. In the first foraging trip, the distance is far from the second trip, because the mobile robots did not have any information of the location power station. Whenever mobile robots got the information, they memorized the location and shared it to other mobile robots. Then for next trips, the mobile robots went to power station directly.

Average of foraging distance by mobile robots in the environment without knowledge sharing shows the high value compared to the mobile robot with knowledge sharing environment. The lowest foraging distance of mobile robot in environment without knowledge sharing is around 133 m compared to the mobile robots in knowledge sharing which is around 39 m. The difference in the foraging distance is around 4 times.

The average of foraging distance in knowledge sharing environment shows that the mobile robots that forage without knowledge for the first foraging trip are higher than the mobile robots that forage with knowledge (see Figure 14(a)). Mobile robots with ID R01, R02, R08, and R09 had to travel further compared to others that already got the knowledge and had foraged more than 30 m in average. Meanwhile mobile robots that already received the information from other robots had traveled less than 30 m.

Meanwhile, in the standard deviation, the mobile robots in environment without knowledge sharing show high variance compared to the mobile robots in environment with knowledge sharing. As a result of mobile robots with knowledge sharing, R01, R02, R08, and R09 have high variance compared to others as they forage without knowledge during the first foraging trip, while others already had the knowledge of power station.

## 5. Discussion and Conclusion 

Based on all results from the measuring parameters in the foraging behavior, decrease in the time consumption, power consumption, and traveling distance is shown. As for time consumption in the foraging area, the applied algorithm to mobile robot system had reduced almost 85% to 89% compared to the nonsharing environment. Meanwhile in the power consumption in the foraging area, the mobile robots with knowledge sharing have been reduced around 78% to 88% compared to mobile robots without knowledge sharing. And finally, the traveling distance in the foraging also reduced around 77% to 84%. The reducing of these parameters in the foraging mobile robots for recharging their battery will increase the time utilization, the power utilization, and the working distance of mobile robot in the working area. The longer the mobile robots in the working area with more power are being utilized and the longer the distance had been traveled to work, this would increase the work utilization in the mobile robot.

This work had identified the algorithm and environment inspired by honeybees system which is applied to multimobile robots by reducing the time consumption, power consumption, and traveling distance during the foraging behavior. Even though the threshold of the behavior of work and foraging is defined by a static threshold and the foraging behavior is random, the three parameters are still reduced with high value. This method has optimized the time consumption, power consumption, and traveling distance in mobile robots by reducing them in foraging area and increasing them in working area.

This system can be improved in the future by restructuring the methods of the system, for example, restructuring the threshold between working and foraging behavior. The threshold could be adaptive in order to maximize the energy in working area for mobile robots that already have the knowledge. Other methods are equipping the mobile robots with the path identification, which means that mobile robots know the path that they already go through. Another matter that also needs to be concerned is the numbers of mobile robots in the environment. This could prevent the unwanted mobile robot that could increase the crowdedness environment.

## Figures and Tables

**Table 1:** Division of labor for honeybees [32, 33].

Period	Day	Stage
First	1-2	Cleaning blood cells
Second	3–9	Tending larvae
Third	10–16	Construction
Fourth	17–20	Guarding the hive
Fifth	21 and above	Foraging food

**Table 2:** Threshold energy of behavior mobile robot.

Remaining energy	Behaviors
*E*_*w*_⩾*E*_th_	Robot's worker
*E*_*f*_ < *E*_th_	Robot's forager

**Table 3:** Illustration of code used in the message format.

Number	Reference	Characters number	Example
1	Location information	**F**: knowledge got by itself	**F**
		**C**: nonknowledge	
		**A**: knowledge got from other robots	
2	Own Robot ID	Numbers	**1**, **2**, **3**, **…**
3	Separator mark	Robot ID separate mark	**$**
4	Opponent robot ID	Numbers	**1**, **2**, **3**, **…**
5	Separator mark	Robots ID separate mark	**$**
6	Coordinate-*x *	Floating number	**2.345**
7	Separator mark	Coordinate-*x* separate mark	;
8	Coordinate-*y *	Floating number	**2.345**
9	End mark of message	Mark of message end	**!**

## References

[1] Mondada F, Pettinaro GC, Guignard A (2004). Swarm-bot: a new distributed robotic concept. *Autonomous Robots*.

[2] Schmickl T, Crailsheim K Trophallaxis among swarm-robots: a biologically inspired strategy for swarm robotics.

[3] Schmickl T, Crailsheim K, Sahin E, Spears W, Winfield A (2007). A navigation algorithm for swarm robotics inspired by slime mold aggregation. *Swarm Robotics*.

[4] Daily Mail Reporter Nuke robots find conditions inside stricken Japanese power plant are ‘sauna-like’. http://dailymail.co.uk/news/article-1378545/Fukushima-Daiichi-nuclear-power-plant-Robots-conditions-inside-sauna-like.html.

[5] Matarić MJ (1995). Issues and approaches in the design of collective autonomous agents. *Robotics and Autonomous Systems*.

[6] Parker LE (1998). ALLIANCE: an architecture for fault tolerant multirobot cooperation. *IEEE Transactions on Robotics and Automation*.

[7] Simmons R, Singh S, Hershberger D, Ramos J, Smith T First results in the coordination of heterogeneous robots for large-scale assembly.

[8] Correll N (2007). *Coordination schemes for distributed boundary coverage with a swarm of miniature robots: synthesis, analysis and experimental validation [Ph.D. thesis]*.

[9] Dorigo M, Floreano D, Maria Gambardella L (2011). Swarmanoid: a novel concept for the study of heterogeneous robotic swarms.

[10] Correll N, Martinoli A (2007). A challenging application in swarm robotics: the autonomous inspection of complex engineered structures. *Bulletin of the Swiss Society For Automatic Control*.

[11] Arvin F, Samsudin K, Rahman Ramli A (2009). Development of a miniature robot for swarm robotic application. *International Journal of Computer and Electrical Engineering*.

[12] Faisul Arif A, Ramli AR, Samsudin K, Hashim SJ Energy management in mobile robotics system based on biologically inspired honeybees behavior.

[13] Beni G, Robotics S (2005). From swarm intelligence to swarm robotics. *Erol Sahin and William Spears*.

[14] Mondada F, Pettinaro GC, Kwee I, Hemelrijk CK, Bonabeau E SWARM-BOT: A swarm of autonomous mobile robots with self- assembling capabilities.

[15] Yoshida E, Yamamoto M, Arai T, Ota J, Kurabayashi D Design method of local communication area in multiple mobile robot system.

[16] Birk A, Condea C, Noda I, Jacoff A, Bredenfeld A, Takahashi Y (2006). Mobile robot communication without the drawbacks of wireless networking. *RoboCup Robot Soccer World Cup IX*.

[17] Oh S, Zelinsky A, Taylor K Autonomous battery recharging for indoor mobile robots.

[18] Silverman MC, Nies D, Jung B, Sukhatme GS Staying alive: a docking station for autonomous robot recharging.

[19] Dung Ngo T, Schioler H A truly autonomous robotic system through self maintained energy.

[20] Wenguo Liu WL, Winfield AFT, Jin Sa JS, Jie Chen JC, Lihua Dou LD (2007). Towards energy optimization: emergent task allocation in a swarm of foraging robots. *Adaptive Behavior*.

[21] Mondada F, Bonani M E-puck—EPFL Education robot. http://www.e-puck.org.

[22] Gerkey B, Vaughan RT, Howard A The player/stage project: tools for multirobot and distributed sensor systems.

[23] Michel O (2004). Webotstm: professional mobile robot simulation. *International Journal of Advanced Robotic Systems*.

[24] Vaughan RT (2008). Massively multi-robot simulation in stage. *Swarm Intelligence*.

[25] Cassinis R, Tampalini F, Bartolini P, Fedrigotti R (2005). Docking and charging system for autonomous mobile robots.

[26] Ngo TD, Schiøler H Sociable mobile robots through self-maintained energy.

[27] Couture-Beil A, Vaughan RT Adaptive mobile charging stations for multi-robot systems.

[28] Karaboga D, Basturk B (2007). A powerful and efficient algorithm for numerical function optimization: artificial bee colony (ABC) algorithm. *Journal of Global Optimization*.

[29] Ranjan Sahoo R, Rakshit P, Haidar MT, Swarnalipi S, Balabantaray Bk, Mohapatra S (2011). Navigational path planning of multi-robot using honey bee mating optimization algorithm (hbmo). *International Journal of Computer Applications*.

[30] Ma Q, Lei X (2010). Dynamic path planning of mobile robots based on abc algorithm. *Artificial Intelligence and Computational Intelligence*.

[31] Bhattacharjee P, Rakshit P, Goswami I, Konar A, Nagar AK Multi-robot path-planning using artificial bee colony optimization algorithm.

[32] Yahya H (2007). *The Miracle of Honey-Bees*.

[33] Fahrbach SE, Robinson GE (1995). Behavioral development in the honey bee: toward the study of learning under natural conditions. *Learning & Memory*.

[34] von Frisch K (1973). Decoding the language of the bee. *Nobel Lecture*.

[35] Kernbach S Jasmine Open-source micro-robotic project. http://www.swarmrobot.org/index.html.

[36] Kernbach S, Thenius R, Kernbach O, Schmickl T (2009). Re-embodiment of honeybee aggregation behavior in an artificial micro-robotic system. *SAGE Adaptive Behavior*.

[37] Caprari G, Siegwart R Mobile micro-robots ready to use: alice.

[38] Egerstedt M, Hu X (2001). Formation constrained multi-agent control. *IEEE Transactions on Robotics and Automation*.

[39] Arvin F, Samsudin K, Ramli AR A short-range infrared communication for swarm mobile robots.

